# Supplementary material for: Effects of Piper betle L. Extract and Allelochemical Eugenol on Rice and Associated Weeds Germination and Seedling Growth
Source: Plants (Basel). 2022 Dec 5;11(23):3384. doi: 10.3390/plants11233384 (PMC9738586; doi:10.3390/plants11233384)
Supplement: Supplementary file 1 [file plants-11-03384-s001.zip › plants-2063439-supplementary.pdf]

Supplementary data

Table S1. Phenylpropanoid constituents in the BE detected by GC-MS

Peak	RT (min)	compounds	% of total
1	9.5	chavicol	0.15
2	10.7	chavicol acetate	1.14
3	11.1	isoeugenol	1.74
4	11.5	methyleugenol	0.05
5	13.1	eugenol acetate	16.18
6	14.5	4-allyl-1,2-diacetoxy benzene	49.68

Table S2. Effects of BE on seeds germination of tested plants by means of paper-plate assay.

Plant samples	Betel extract (mg/mL)	Germination (%)
Rice	0	99 ± 1.2 [a]
	0.25	98 ± 0.0 [a]
	0.5	96 ± 0.0 [a]
	1	74 ± 4.0 [b]
	2	14 ± 2.0 [c]
Swollen finger grass	0	93 ± 6.1 [a]
	0.05	8 ± 3.5 [b]
	0.1	0 ± 0.0 [c]
	0.2	0 ± 0.0 [c]
False daisy	0	97 ± 2.3 [a]
	0.125	93 ± 1.2 [a]
	0.25	57 ± 4.2 [b]
	0.5	11 ± 4.2 [c]
	1	0 ± 0.0 [d]
Barnyard grass	0	100 ± 0.0 [a]
	0.25	96 ± 2.0 [b]
	0.5	96 ± 2.0 [b]
	1	20 ± 2.0 [c]
	2	0 ± 0.0 [d]
Weedy rice	0	99 ± 2.3 [a]
	1	95 ± 6.1 [a]
	2	13 ± 8.3 [b]
	3	0 ± 0.0 [c]

Values are means ± SD of triplicate sets (n > 100). Different superscript letters indicate statistically significant differences of germination among different BE concentrations at $p < 0.05$

Table S3. Effects of BE on root and shoot length of 7DAG seedlings by means of paper-plate assay.

Plant samples	Betel extract (mg/mL)	Length (cm)		Root/Shoot ratio	R/S ratio (%)
		Root	Shoot		
Rice	0.00	6.2 ± 1.8 [a]	3.3 ± 0.7 [a]	1.9	100
	0.25	4.6 ± 1.3 [b]	2.7 ± 0.8 [b]	1.7	89
	0.50	2.9 ± 0.9 [c]	2.4 ± 0.8 [b]	1.2	63
	1.00	0.6 ± 0.3 [d]	1.8 ± 0.8 [c]	0.3	16
	2.00	0.0 ± 0.0 [d]	0.8 ± 0.5 [d]	0.0	0
Swollen finger grass	0.00	1.4 ± 0.2 [a]	0.7 ± 0.1 [a]	2.0	100
	0.05	1.2 ± 0. 2 [b]	0.8 ± 0.1 [a]	1.5	75
	0.10	0.0 ± 0.0 [c]	0.0 ± 0.0 [b]	0.0	0
	0.20	0.0 ± 0.0 [c]	0.0 ± 0.0 [b]	0.0	0
False daisy	0.00	1.5 ± 0.5 [a]	0.6 ± 0.2 [a]	2.5	100
	0.125	0.8 ± 0.3 [b]	0.5 ± 0.1 [b]	1.6	64
	0.25	0.3 ± 0.1 [c]	0.4 ± 0.1 [c]	0.7	28
	0.50	0.0 ± 0.0 [d]	0.3 ± 0.1 [d]	0.0	0
	1.00	0.0 ± 0.0 [d]	0.0 ± 0.0 [e]	0.0	0
Barnyard grass	0.00	2.4 ± 1.0 [a]	2.9 ± 0.6 [a]	0.8	100
	0.25	2.2 ± 0.7 [a]	3.0 ± 0.6 [a]	0.9	113
	0.50	0.3 ± 0.1 [b]	2.8 ± 0.6 [a]	0.2	25
	1.00	0.0 ± 0.0 [b]	0.4 ± 0.3 [b]	0.0	0
	2.00	0.0 ± 0.0 [b]	0.0 ± 0.0 [c]	0.0	0
Weedy rice	0.00	5.6 ± 1.7 [a]	3.6 ± 1.2 [a]	1.5	100
	1.00	0.5 ± 0.3 [b]	2.1 ± 0.7 [b]	0.2	13
	2.00	0.0 ± 0.0 [b]	0.4 ± 0.2 [c]	0.0	0
	3.00	0.0 ± 0.0 [b]	0.0 ± 0.0 [c]	0.0	0

Values are means ± SD of triplicate sets (n > 100). Different superscript letters indicate statistically significant differences of length among different BE concentrations at $p < 0.05$.

Table S4. Effects of EU on seeds germination of tested plants by means of paper-plate assay.

Plant samples	Eugenol (mg/mL)	Germination (%)
Rice	0	99 ± 2.3 [a]
	0.08	95 ± 2.3 [a]
	0.16	96 ± 0.0 [a]
	0.32	77 ± 4.2 [b]
	0.64	0 ± 0.0 [c]
Swollen finger grass	0.00	93 ± 5.8 [a]
	0.02	80 ± 10.6 [ab]
	0.04	68 ± 6.9 [b]
	0.08	0 ± 0.0 [c]
False daisy	0.00	95 ± 2.3 [a]
	0.04	91 ± 7.6 [ab]
	0.08	86 ± 5.3 [ab]
	0.16	91 ± 1.2 [b]
	0.32	0 ± 0.0 [c]
Barnyard grass	0.00	97 ± 4.6 [a]
	0.08	95 ± 2.3 [a]
	0.16	92 ± 4.0 [a]
	0.32	2 ± 2.0 [b]
Weedy rice	0.00	100 ± 0.0 [a]
	0.08	100 ± 0.0 [a]
	0.16	100 ± 0.0 [a]
	0.32	100 ± 0.0 [a]
	0.64	0 ± 0.0 [b]

Values are means ± SD of triplicate sets (n >100). Different superscript letters indicate statistically significant differences of germination among different EU concentrations at $p < 0.05$

Table S5. Effects of EU on root and shoot length of 7DAG seedlings by means of paper-plate assay.

Plant samples	Eugenol (mg/mL)	Length (cm)		Root/Shoot ratio	R/S ratio (%)
		Root	Shoot		
Rice	0.00	3.8 ± 0.5 [a]	1.6 ± 0.3 [a]	2.4	100
	0.08	4.3 ± 0.7 [b]	1.9 ± 0.6 [b]	2.2	92
	0.16	2.7 ± 0.7 [c]	1.0 ± 0.3 [c]	2.7	112
	0.32	0.1 ± 0.1 [d]	0.3 ± 0.1 [d]	0.4	17
	0.64	0.0 ± 0.0 [d]	0.0 ± 0.0 [e]	0.0	0
Swollen finger grass	0.00	2.1 ± 0.2 [a]	1.2 ± 0.0 [a]	1.8	100
	0.02	2.2 ± 0.2 [a]	1.2 ± 0.0 [a]	1.8	104
	0.04	0.6 ± 0.1 [b]	0.4 ± 0.1 [b]	1.5	86
	0.08	0.0 ± 0.0 [c]	0.0 ± 0.0 [c]	0.0	0
False daisy	0.00	2.3 ± 0.4 [a]	0.7 ± 0.2 [a]	3.3	100
	0.04	1.6 ± 0.5 [b]	0.6 ± 0.2 [a]	2.6	79
	0.08	0.6 ± 0.2 [c]	0.7 ± 0.3 [a]	0.9	26
	0.16	0.1 ± 0.0 [d]	0.3 ± 0.1 [b]	0.4	12
	0.32	0.0 ± 0.0 [d]	0.0 ± 0.0 [c]	0.0	0
Barnyard grass	0.00	2.8 ± 1.7 [a]	3.0 ± 0.1 [a]	0.9	100
	0.08	2.9 ± 0.1 [a]	2.8 ± 0.0 [a]	1.0	111
	0.16	0.8 ± 0.2 [b]	1.4 ± 0.1 [b]	0.6	61
	0.32	0.0 ± 0.0 [c]	0.0 ± 0.0 [c]	0.0	0
Weedy rice	0.00	6.1 ± 0.2 [a]	5.7 ± 0.0 [a]	1.1	100
	0.08	5.8 ± 0.3 [a]	5.0 ± 0.2 [b]	1.1	108
	0.16	3.0 ± 0.4 [b]	5.2 ± 0.3 [b]	0.6	53
	0.32	2.1 ± 0.1 [c]	2.6 ± 0.1 [c]	0.8	76
	0.64	0.0 ± 0.0 [d]	0.0 ± 0.0 [d]	0.0	0

Values are means ± SD of triplicate sets (n >100). Different superscript letters indicate statistically significant differences of length among different EU concentrations at $p < 0.05$.

Table S6. Effect of betel extract on rice seedling root growth and development by means of in-gel assay.

Betel extract (mg/mL)	PR Length (cm)	CR Number	CR length (cm)	LR density
0	5.8 ± 0.4^{a}	3.0 ± 0.0^{a}	2.5 ± 0.2^{a}	11.2 ± 1.7^{a}
0.025	5.3 ± 0.4^{a}	3.0 ± 0.0^{a}	2.4 ± 0.3^{a}	8.7 ± 2.4^{a}
0.05	3.1 ± 0.1^{b}	2.9 ± 0.1^{a}	2.1 ± 0.0^{b}	5.6 ± 2.9^{ab}
0.1	1.3 ± 0.2^{c}	2.2 ± 0.3^{b}	0.5 ± 0.3^{c}	2.7 ± 2.3^{b}
0.2	0.2 ± 0.1^{d}	0.0 ± 0.0^{c}	0.0 ± 0.0^{d}	0.0 ± 0.0^{b}

Values are means ± SD of triplicate sets (n > 30). Different superscript letters indicate statistically significant differences of values among different BE concentrations at $p < 0.05$.
PR, primary root; CR, crown root; LR, lateral root

Table S7. Effect of eugenol on rice seedling root growth and development by means of in-gel assay.

Eugenol (mg/mL)	PR Length (cm)	CR Number	CR length (cm)	LR density
0	5.3 ± 0.8 [a]	3.0 ± 0.0 [a]	2.3 ± 0.6 [a]	9.0 ± 0.5 [a]
0.025	3.2 ± 0.4 [b]	2.9 ± 0.2 [a]	1.4 ± 0.6 [ab]	9.7 ± 1.0 [a]
0.05	2.9 ± 0.2 [b]	2.4 ± 0.1 [b]	0.8 ± 0.4 [bc]	4.7 ± 2.0 [b]
0.1	1.6 ± 0.0 [c]	1.0 ± 0.0 [c]	0.3 ± 0.1 [c]	0.0 ± 0.0 [c]
0.2	0.3 ± 0.1 [d]	0.0 ± 0.0 [d]	0.0 ± 0.0 [c]	0.0 ± 0.0 [c]

Values are means ± SD of triplicate sets ($n > 30$). Different superscript letters indicate statistically significant differences of values among different EU concentrations at $p < 0.05$. PR, primary root; CR, crown root; LR, lateral root
